# *Fiat* or *Bona Fide* Boundary—A Matter of Granular Perspective

**DOI:** 10.1371/journal.pone.0048603

**Published:** 2012-12-12

**Authors:** Lars Vogt, Peter Grobe, Björn Quast, Thomas Bartolomaeus

**Affiliations:** 1 Institut für Evolutionsbiologie und Ökologie, Universität Bonn, Bonn, Germany; 2 Forschungsmuseum Alexander Koenig Bonn, Bonn, Germany; University of South Florida College of Medicine, United States of America

## Abstract

**Background:**

Distinguishing *bona fide* (i.e. natural) and *fiat* (i.e. artificial) physical boundaries plays a key role for distinguishing natural from artificial material entities and is thus relevant to any scientific formal foundational top-level ontology, as for instance the Basic Formal Ontology (BFO). In BFO, the distinction is essential for demarcating two foundational categories of material entity: object and *fiat* object part. The commonly used basis for demarcating *bona fide* from *fiat* boundary refers to two criteria: (i) intrinsic qualities of the boundary bearers (i.e. spatial/physical discontinuity, qualitative heterogeneity) and (ii) mind-independent existence of the boundary. The resulting distinction of *bona fide* and *fiat* boundaries is considered to be categorial and exhaustive.

**Methodology/Principal Findings:**

By referring to various examples from biology, we demonstrate that the hitherto used distinction of boundaries is not categorial: (i) spatial/physical discontinuity is a matter of scale and the differentiation of *bona fide* and *fiat* boundaries is thus granularity-dependent, and (ii) this differentiation is not absolute, but comes in degrees. By reducing the demarcation criteria to mind-independence and by also considering dispositions and historical relations of the bearers of boundaries, instead of only considering their spatio-structural properties, we demonstrate with various examples that spatio-structurally *fiat* boundaries can nevertheless be mind-independent and in this sense *bona fide*.

**Conclusions/Significance:**

We argue that the ontological status of a given boundary is perspective-dependent and that the strictly spatio-structural demarcation criteria follow a static perspective that is ignorant of causality and the dynamics of reality. Based on a distinction of several ontologically independent perspectives, we suggest different types of boundaries and corresponding material entities, including boundaries based on function (locomotion, physiology, ecology, development, reproduction) and common history (development, heredity, evolution). We argue that for each perspective one can differentiate respective *bona fide* from *fiat* boundaries.

## Introduction

Data integration, data comparability, and the development of data and metadata standards are becoming more and more important in times of increased communication via the World Wide Web and an increasing importance of online databases in academia. Ontologies and other techniques of the Semantic Web thereby play a key role for reliably communicating and managing data within and between databases. This also applies to the life sciences, for which different ontologies for different domains and different purposes already exist (cf., BioPortal; http://bioportal.bioontology.org). Unfortunately, these ontologies often differ considerably [1]–[3], resulting in incompatibilities and inconsistencies between the contents of the databases that use them and in how these contents are being represented in them. Therefore, in order to achieve common data and metadata standards, ontologies must be standardized as well. Formal top-level ontologies [1], [4], as for instance the Descriptive Ontology for Linguistic and Cognitive Engineering (DOLCE) or the Basic Formal Ontology (BFO), play a key role in this respect. They are intended to provide domain- and purpose-independent theories within a formal framework of axioms and definitions for most general terms and concepts, which can be used as a top-level template and formal framework for developing domain reference ontologies and terminology-based application ontologies [1]–[3], [5].

Among many other things, formal top-level ontologies must provide explicit and unambiguous definitions for top-level categories of foundational types of material entity, which scientists from all domains and research interests can agree upon. Smith [6], [7] introduced the distinction of two foundational types of boundaries of physical entities, on which BFO's top-level distinction between *fiat* and *bona fide* material entities is based on:

1. ***Bona fide*** **boundaries:** natural or *mind-independent* boundaries [7], [8], which are physical boundaries in the things themselves that exist independently from human perception [6]–[10].
2. ***Fiat*** **boundaries:** artificial (i.e. artifact of cognition) or *mind-dependent* boundaries, which are non-physical boundaries that depend on human decision and thus are the products of mental activities [6]–[9].

The BFO calls the two corresponding top-level categories of material entity ‘object’ and ‘fiat object part’, respectively (http://www.ifomis.org/bfo; [11]).

In their very general meaning of *mind-dependent* and *mind-independent*, however, the two attributes ‘*fiat*’ and ‘*bona fide*’ can be applied in various contexts and are not restricted to boundaries of physical entities. Thus, one can even talk about *fiat* concepts in a conceptual sense, or about perceptual, ecological, geometrical, legal, administrative, political and linguistic fiats (e.g., [7], [8], [12]). Obviously, the distinction of *fiat* and *bona fide* goes beyond the physical realm and is very general. The categories of *bona fide* and *fiat* boundary, when they are used in the context of physical boundaries, however, are very specific and depend on specific spatio-structural properties and thus properties that are intrinsic to the physical entity the boundary bounds (e.g. [6]–[9]).

Material entities, however, possess additional natural properties, besides their spatio-structural properties, as for instance functional dispositions or historical relations. Strictly confining the application of the attributes ‘*bona fide*’ and ‘*fiat*’ for boundaries to the presence of specific spatio-structural properties does not necessarily follow from the general notion of *fiatness* and *bona fideness*, since not only spatio-structural properties but also dispositions or historical relations can exist independently of human cognitive acts. As a consequence, dispositions and historical relations can be differentiated into mind-dependent and mind-independent ones as well. What is the reason for not referring to dispositions and historical relations for distinguishing *fiat* and *bona fide* boundaries and instead restricting them to spatio-structural properties? Moreover, considering the importance of boundaries for the demarcation of top-level categories of material entity, why do we rest the decision of whether a material entity is an object or a *fiat* object part exclusively on spatio-structural grounds? Is the distinction between *bona fide* and *fiat* boundary only categorial and absolute within this purely spatio-structural context?

We start with discussing the distinction of *bona fide* and *fiat* boundaries and their relation to the distinction of *bona fide* objects and *fiat* object parts within the spatio-structural context. By focusing on some borderline cases we discuss whether the distinction is categorial or rather granularity-dependent. Then we take a closer look at biological entities and aspects of continuity and connectedness characteristic of them. We argue that in the biological domain the granularity-dependence of boundaries can be found in all levels of granularity. We point out the role of *bona fide* landmarks for recognizing *fiat* boundaries, concluding that both *fiatness* of boundaries and *fiatness* of material entities comes in degrees. Since reality is dynamic and biological entities actively participate in many biological processes (i.e. evolution, embryogenesis, physiology, etc.), we argue that the criteria used for distinguishing *fiat* from *bona fide* boundaries and *fiat* from *bona fide* entities, respectively, must not only consider intrinsic spatio-structural qualities but also the dispositions and historical relations of material entities. By providing adequate examples from biology, we demonstrate that in many cases material entities that are demarcated by spatio-structurally *fiat* boundaries are nevertheless *bona fide* units in the sense that their existence as natural units is in fact mind-independent. Thus, it can be demonstrated that the differentiation into mind-dependent and mind-independent boundaries is not only granularity-dependent in a spatio-structural sense, but also perspective-dependent. We distinguish a structural from a functional and a historical perspective for the biological domain, thereby contrasting structural anatomy (*form*) with functional anatomy and historical/evolutionary anatomy. Finally, we draw the consequences for ontology design and formal top-level ontologies in terms of the requirement for integrating several perspective-dependent taxonomies of top-level categories of material entity and their relations to each other.

## Results

### 2.1 The Spatio-Structural Notion of Top-Level Categories of Boundary and Material Entity

The ontological relation between types of boundaries and types of material entities is strong, because the existence of boundaries depends on the higher-dimensional entities they bound, i.e. their hosts [7], [9], [13]. Traditionally, a *bona fide* material object is characterized as an entity that extents in space and that can be demarcated clearly and unambiguously from its respective environment (i.e. its complement—the universe without this particular material entity). It possesses a single continuous outer boundary, usually referred to as its surface, which symmetrically demarcates the object from its complement and vice versa [9]. Because the boundary belongs to its material host and not to the complement, the respective material object is considered to be closed and its complement to be open. These outer boundaries are called *bona fide* boundaries [6] (also called *natural boundary*, [14]) and can be demarcated on grounds of *“some interior physical discontinuity or some qualitative heterogeneity among the parts of the object (some sharp gradient of material constitution, color, texture, electric charge, etc.)”* [8]. *Bona fide* boundaries are physical boundaries in the things themselves and *“exist independently of all human cognitive acts – they are a matter of qualitative differentiations or discontinuities in the underlying reality”* [7]. The surface of your skin and the surface of an apple in a fruit basket represent examples of *bona fide* boundaries of material objects.

Because every material entity consists of divisible matter, it can be divided spatially along inner boundaries into its constitutive parts. Inner boundaries are not necessarily *bona fide* boundaries, because they do not necessarily have to follow any physical discontinuity or qualitative heterogeneity. A boundary that is not *bona fide* is called a *fiat boundary* (also called *artificial boundary*, [14]):

*“the demarcations induced by fiat boundaries are not grounded in any intrinsic features of the underlying reality, and correspond only to cognitive phenomena such as those induced by our use and understanding of political maps and cadastral surveys”* [9].

According to Smith and Varzi [9], *“the categorial distinction between fiat and bona fide boundaries is absolute”*, meaning that no instance of the type ‘*fiat* boundary’ instantiates the type ‘*bona fide* boundary’—their extensions do not overlap. *Fiat* boundaries are considered to be non-physical boundaries that exclusively depend on acts of human decision:

*“we cannot directly see fiat boundaries”* [15] and they *“are the products of our mental and linguistic activity, and of associated conventional laws, norms and habits”* [8].

In other words, *fiat* boundaries are arbitrarily imposed [16], not grounded in the autonomous mind-independent world [14], but are *“human-demarcation-induced”* [7], [9] boundaries. They *“are in a sense potential in that they do not actually separate anything from anything—they do not mark any actual discontinuity”* [9]. Instead, they represent boundaries, which owe their existence to conventional laws, agreements, political decrees and habits, and they do not separate anything in reality [7]–[10]. The moment one cuts an object along one of its *fiat* boundaries, one divides it into two new objects and the formerly *fiat* boundary of the original object would be gone and, so to speak, replaced by two newly created *bona fide* boundaries.

The Equator, but also the inner boundary demarcating your right thumb from the rest of your body, are good examples for *fiat* boundaries. A *fiat* inner boundary of a *bona fide* object constitutes the *fiat* outer boundary of one of the object's *fiat* parts. Contrary to a *bona fide* boundary, a *fiat* boundary is shared by the two *fiat* parts it demarcates (i.e. each part possesses its own *fiat* boundary, but the two boundaries are considered to *coincide*; see [9], [10]).

The differentiation between *bona fide* and *fiat* boundaries is important if one wants to distinguish between the two basic top-level categories of material entity, *bona fide objects* and *fiat objects parts*. *Bona fide* objects are bound completely by a continuous *bona fide* outer boundary, whereas *fiat* object parts are limited by at least one *fiat* boundary [6]–[10]. Accordingly, *bona fide* objects are assumed to exist independent of human cognitive activities. *Fiat* object parts, on the other hand, owe their existence to the recognition and the establishment of *fiat* boundaries through partitioning activities. These are based on decisions or conceptually guided demarcations that demand a symbolic, reflective or linguistic capacity on the part of a human being [7]. The resulting ontology of *fiat* entities is thus concept-dependent and accessible only to linguistic human beings. Therefore, following this notion of boundaries, one has to conclude that whereas your body and an apple in a fruit basket exist independently of any human cognitive acts, boundaries delimiting an apple on a tree, the thumb of your right hand, your right upper arm, mesodermal tissue and the active center of an enzyme would not. Instead, their existence would depend on the cognitive activity of a human agent.

### 2.2 Borderline Cases

#### 2.2.1 Vagueness and Indeterminate Boundaries—Are Boundaries always Crisp?

When thinking of physical boundaries (as contrasted with e.g. political, legal or linguistic boundaries) we usually think of clear-cut lines separating one entity from its environment. In case of three-dimensional entities the boundaries are surfaces and thus entities of two dimensionality—they are not three-dimensional bodies themselves. And in case of surfaces the physical boundaries are lines and not regions. One could conclude that, regardless of whether boundaries are *fiat* or *bona fide*, they are necessarily *crisp* in this respect that they are always of a dimensionality one less than their hosts' dimensionality.

When dealing with real entities and their actual boundaries, however, a supposedly different picture emerges. In some cases, like for instance the boundaries of many geographical objects such as deserts, dunes, or the Caribbean Sea, it seems that a given entity cannot be delineated by crisp boundaries—we have troubles to identify a single surface that demarcates a desert, dune, or the Caribbean Sea. Instead, they are delineated by *border zones*, i.e. by boundary-like *regions*, which are indeterminate to some degree [6]–[10], [17]. Consequently, we would have to distinguish between *crisp* (i.e. sharp) and *indeterminate* (i.e. fuzzy, vague) boundaries [18], [19], with crisp boundaries always possessing a dimensionality one lower than their host. Thus, one could ask whether an indeterminate boundary, on the other hand, always shares the dimensionality with its host.

An indeterminate boundary could be interpreted as the region in which an ontologically crisp boundary must be located, but we simply cannot narrow down its actual location. In this case, the indeterminacy would represent a conceptual issue that is owed to linguistic or epistemological problems instead of ontological ones (cf. [14]). Thus, we must distinguish an *ontological* from an *epistemic-conceptual* interpretation of the indeterminacy of some physical boundaries. The epistemic-conceptual interpretation of the indeterminacy problem, which is favored by various authors (e.g. [7], [8], [10], [17], [20], [21]), shifts the indeterminacy problem to a supposed vagueness of the respective concepts, instead of looking for an ontological reason (e.g. quantum mechanics). They argue that indeterminate boundaries can be defined in principle, but they cannot be determined precisely. Interestingly, the examples that are commonly used in this context usually refer to *fiat* entities. Therefore, one could also argue that the vagueness of the respective boundaries is owed to their *fiat* nature and thus to the *fiatness* of the entities involved. It is thus the vagueness of the corresponding concepts themselves, resulting from their mind-dependent conceptual nature, that is responsible for the indeterminacy (e.g. [8], [17]). Examples are for instance entities that are heterogeneously composed, with two clearly distinguishable poles that are not sharply delimited from each other by a crisp boundary. Instead, they are bound by a region in which they merge seamlessly, as it is often the case with different and overlapping chemical gradients in biological objects (e.g. the polarity of a blastula during embryogenesis, with the animal pole and the vegetale pole).

The indeterminacy of time dependent boundaries, like for instance coastlines or river banks, is another example for epistemic-conceptually indeterminate boundaries. Coastlines are shifting borders, and since they are crisp and *bona fide* at any given moment in time, their indeterminacy can be attributed to the problem of the time dependence of their actual location [9]. Their indeterminacy does not pose fundamental problems to ontology design, since the vagueness is just an epiphenomenon of the dynamics of reality.

Thus, as long as indeterminate boundaries can be restricted to *fiat* entities or attributed to the dynamics of reality, they do not pose fundamental problems to ontology design, as they nicely match with the basic categorial distinction of *bona fide* and *fiat* boundaries and the accompanied basic categorial distinction of *bona fide* objects and *fiat* entities respectively.

But are *bona fide* boundaries really always crisp at a given moment in time? The distinction of what counts as physical discontinuity or qualitative heterogeneity on the one hand and physical continuity and qualitative homogeneity on the other hand, is not crisp. There are cases of ontological indeterminacy—*bona fide* boundaries that are less *bona fide* than others, so to speak.

It cannot be denied that discontinuities come in various degrees of abruptness. The cutting edge of a sharp knife comes more abruptly than the edge of the white cliffs of Dover. From a purely perceptual point of view, the edges of the letters that appear on your screen while typing an email are crisper than the color changes in a rainbow. Obviously, **discontinuity is a matter of scale and therefore of granularity**. With increasing resolution spatial boundaries of physical entities become increasingly fuzzy. This results in what has been called the *Problem of the Many* [22], [23], which Lewis characterizes as follows:

*“Think of a cloud — just one cloud, and around it a clear blue sky. Seen from the ground, the cloud may seem to have a sharp boundary. Not so. The cloud is a swarm of water droplets. At the outskirts of the cloud, the density of the droplets falls off. Eventually they are so few and far between that we may hesitate to say that the outlying droplets are still part of the cloud at all; perhaps we might better say only that they are near the cloud. But the transition is gradual. Many surfaces are equally good candidates to be the boundary of the cloud. Therefore many aggregates of droplets* (…) *are equally good candidates to be the cloud. Since they have equal claim, how can we say that the cloud is one of these aggregates rather than another? But if all of them count as clouds, then we have many clouds rather than one. And if none of them count, each one being ruled out because of the competition from the others, then we have no cloud. How is it, then, that we have just one cloud? And yet we do.”* [23]

Whereas one could argue that the *Problem of the Many* represents a purely linguistic and epistemological problem of referencing and the use of language rather than an ontological problem, it nonetheless results from the vagueness or fuzzyness of boundaries. The relevance of this problem becomes apparent when considering that at the subatomic level, any physical entity resolves into a swarm of subatomic particles, of which no clearly determinable boundary can be specified—the location and shape of the outer surface of a material entity involves some degree of arbitrariness at these fine levels of granularity [9], [14], [17]. Thus, at least at very fine levels of granularity, the idea of abrupt physical discontinuities seems to be questionable [8].

At coarser levels of granularity, however, the dichotomy between *fiat* and *bona fide* boundaries and their respective physical entities can be maintained and seems to be a reasonable distinction [8], [9], [17]. From which granularity level onwards this dichotomy applies depends on the entity and cannot be determined universally. In other words, when increasing the resolution some entities turn fuzzy in coarser levels of granularity than others.

Whether the indeterminacy at finer levels of granularity and the *granularity-dependence* of the *bona fideness* of boundaries as such can be solely ascribed to conceptual and epistemological issues, or whether it is evidence for an underlying ontological vagueness, represents an open question.

#### 2.2.2 Continua in Biological Objects and the Distinction of *Fiat* and *Bona Fide* Boundaries—Is this Distinction really Absolute?

According to the above discussed spatio-structural notion of boundaries, the question whether a given boundary is a *bona fide* boundary or a *fiat* boundary only depends on the question of what counts as physical discontinuity or qualitative heterogeneity on the one hand and what as physical continuity and qualitative homogeneity on the other hand. It is the question of where to draw the line between these two conditions. This decision immediately concerns the distinction of objects and *fiat* object parts.

According to its definition in BFO, an object is a material entity that is maximally self-connected and self-contained [11]. This definition draws on a *principle of connectedness* that allows only ‘true’ or ‘false’ as possible values, while at the same time functioning as a *principle of unity* for the entity to be delineated (e.g. [24]). This is insofar problematic, as *connectedness* comes in degrees, which at its turn results from an underlying continuity problem [12], [17] that frequently impedes consistently distinguishing *bona fide* from *fiat* boundaries.

When considering *physical* connectedness, one has to deal with various types of connectedness, ranging from gravitational forces to all kinds of electro-chemical bonds (covalent bonds, ionic bonds, metallic bonds, hydrogen bonds, etc.) and physical connections and junctions (screws, bolts, staples, nails, etc.). Part of the problem is also the fact that what counts as maximally self-connected is granularity-dependent and deals with the problem of how aggregations of *bona fide* objects of finer levels of granularity constitute a single *bona fide* object of a coarser level of granularity—how does an aggregate of individual cells, with each cell having its own *bona fide* boundary, constitute a single multicellular organism at a coarser level of granularity that also possesses its own boundary (cf. [25], [26])?

**Continuity:** Although continuity problems affect all kinds of material entities, they are especially serious in biology. Biological objects are the product of evolution and exhibit a high degree of variability that constitutes a complex network of relations of similarities and differences between all objects of the enlivened nature. Accordingly, biologists have to deal with a continuum of forms and functions that spans a complex morphological property space, in which usually no two objects occupy exactly the same place [12]. This alone poses considerable conceptual problems [27], and many of the concepts used for referring to specific types of entities within this continuum are delineated by *fiat* (cf. *fiat concepts*; [8]). But this problem of delineating different types of entities is not exclusively of conceptual nature: if, due to the high degree of variability, no two cells are identical, any aggregation of cells exhibits qualitative heterogeneity between any two neighboring cells. When distinguishing biological objects above the cellular level one is thus confronted with the question where to draw the line between relevant and irrelevant heterogeneity, since heterogeneity is always present but not always relevant. As a consequence, at supracellular levels of granularity the distinction of *bona fide* and *fiat* concepts is not crisp anymore and involves some fuzziness that cannot be explained by referring to conceptual problems alone. Instead, this fuzziness is the result of an underlying ontological continuum.

**Connectedness:** Another problem in biology is the fact that, except for whole organisms, all anatomical objects of the cellular and supracellular levels of granularity are connected to neighboring anatomical objects via conduits, tunnels, vessels, ducts, nerve cords, intercellular spaces, pores, channels, and junctions (cf. [12], [28]). These connections are products of evolution, are functionally necessary, and are characteristic to complex systems of interacting subsystems, such as multicellular organisms and their parts. As a consequence, anatomical objects possess regions within their otherwise *bona fide* outer boundary that are *fiat* [12]. If the dichotomy of *fiat* and *bona fide* boundaries is absolute, as it has been claimed [9], [10], the respective entities would have to be treated as *fiat* entities. Consequently, no *bona fide* biological objects would exist at levels of granularity coarser than the molecular level, since even organelles exhibit such connections.

Some authors argue, however, that in many cases the fraction of *fiat* boundaries is very small compared to the total outer boundary of anatomical objects and, therefore, can be ignored in these cases (e.g. [28]). The question, then, is what are the criteria on which to decide whether a given portion of *fiat* boundaries can be ignored? How much *fiatness* can be tolerated? And how could this fit with the claimed categorial and thus absolute distinction of *fiat* and *bona fide* boundary?

**Granularity-dependence:** Apparently, regarding continuity and connectedness we are, again, dealing with a granularity-dependence of the *bona fideness* of boundaries. Unlike the *general granularity-dependence* of physical *bona fide* boundaries discussed above, however, in which *all* physical boundaries lose their overall *bona fideness* at very fine levels of granularity and become fuzzy, the *specific granularity-dependence* of biological objects is gradual. For example the liver of a cow: The liver is surrounded by a compact layer of extracellular matrix and the peritoneum, which at its turn surrounds most organs of the trunk. The liver is thus located in a large basal swell of the peritoneum, which almost completely surrounds it. As a consequence, one can easily demarcate the liver from the rest of the cow at first glance by using traditional preparation techniques and inspection by eye. After closer inspection, however, one will realize that the liver is connected to the rest of the body by various blood vessels, bile ducts and nerve cords. Moreover, when using a light microscope, one will realize after preparation that the number of vessels, bile canaliculi and nerve cords is much higher than expected. However, on the light microscopic level of resolution one would still think that the combination of cell membranes of the outer most liver cells provides the liver a continuous *bona fide* boundary that is only interrupted by the lumina and axons of the afore noticed vessels, capillaries and nerve cords. After increasing resolution by using electron microscopy, however, one will see that even the cell membranes themselves do not provide *bona fide* boundaries for their cells, because each membrane is interrupted by gap junctions, which connect the cytoplasm of the liver cells with each other and with the surrounding tissue. As a consequence, the total outer boundary of each liver cell is interrupted by a small fraction of *fiat* boundaries and the fraction of *fiat* boundaries of the total outer boundary of the cow liver is larger than expected by light microscopy. Therefore, at some level of granularity, the *bona fide* total boundary of a supramolecular biological object will turn into a *bona fide* boundary interrupted by very few portions of *fiat* boundaries. When further increasing the resolution, the proportion of *fiat* to *bona fide* boundary often increases as well. This is independent of and qualitatively different from the *general granularity-dependence* of *bona fide* boundaries discussed further above, which only comes into effect at very fine levels of granularity and results in a switch from a *bona fide* total boundary to a *fiat* total boundary.

The *specific granularity-dependence* poses fundamental problems to ontology design in the biomedical domain. Since the connections (i.e. conduits, tunnels, vessels, ducts, nerve cords, intercellular spaces, pores, channels, and junctions) play a fundamental causal role in those biological systems to which they belong, they are of genuine interest to the biomedical domain and cannot be ignored in ontology design. Whereas for most domains the *general granularity-dependence* of the *bona fideness* of boundaries might be unproblematic, since it is usually restricted to levels of granularity that are outside their scope and focus, this is not the case for the *specific granularity-dependence* of biological objects, which is not restricted to the finer levels of granularity. As a consequence, at least the *specific granularity-dependence* of the *bona fideness* of physical boundaries and therefore also the granularity-dependence of the distinction of objects and *fiat* object parts must be accounted for in ontology design in the biomedical domain. For an approach how this can be achieved in principle via using different representations for the same type of entity for different levels of granularity see Vogt *et al.* [26].

#### 2.2.3 *Fiat* Boundaries and *Bona Fide* Landmarks—Spatio-Structural *Fiatness* comes in Degrees

The discussion above demonstrates that any given *bona fide* boundary can be *bona fide* at some coarser level of granularity and *fiat* at finer ones. It seems as if *fiatness*, or *bona fideness* respectively, comes in degrees. This impression is reinforced when considering that the possibility to reliably specify and re-locate any given *fiat* boundary requires some *bona fide* landmarks and coordinates (i.e. *bona fide* parts of the same or lower dimensionality that are used to locate a *fiat* boundary, as for instance the juncture of two blood vessels as a *bona fide* landmark for locating a *fiat* boundary between the two parts of the vessel, the part before and the part after the juncture) or other pragmatic or even scientifically justified criteria [7], [8], [29]. In other words, *fiat* entities are to varying degrees supervenient on *bona fide* objects on finer levels of granularity [7], or some other unambiguously identifiable landmarks. From this follows that, because their specification and re-location involves real properties of the underlying factual materials, *fiat* entities usually owe their existence not exclusively to human *fiat* [10]. These real properties also constrain the range of locations of *fiat* boundaries that are relevant in the scientific discourse.

In its pure and strictest meaning, *fiatness* implies *mind-dependence*, and thus a *fiat* boundary is a boundary that is determined by human *fiat*, lacking any natural indication. *Fiat* boundaries in this sense, however, would be inapplicable in any practical context. Instead, *fiat* boundaries of interest usually rest on:

- threshold values as *fiat* landmarks within a continuous heterogeneous field, the values being based on some legal or otherwise specified convention and agreement, as for instance isobars, the International Date Line, or meters over mean sea level;
- *bona fide* mathematical and topological landmarks, for example the center of mass of a material entity, the upper and lower hemisphere of a rotating sphere, or the saddle point of a curve (cf. [9], [10]);
- *bona fide* landmarks within a homogeneous field that are based on natural units, as for instance the classification of chemical elements based on their characteristic number of units of mass;
- spatio-structural *bona fide* landmarks, as for instance the demarcation of an apple hanging from a tree, which is supervenient on the branching point of the stalk from the branch it is connected to, or the *fiat* boundaries of your right upper arm, which are supervenient on your armpit and elbow that relate to the position and function of your humerus and its associated muscles, all of which are *bona fide* objects themselves;
- the identification of causal subsystems, i.e. spatio-structural parts that actively participate in causal processes, which are characteristic to the subsystem and that play a causal role within the system as a whole, as for instance the apple on a tree as a unit of reproduction, your thumb or your right upper arm as units of locomotion, the mesodermal germ layer as a unit of embryogenesis, or the active center of an enzyme as a physiological (biochemical) unit.

These examples of different types of *fiat* boundaries demonstrate the broad range of degrees of *fiatness* that can be involved when dealing with biological entities, ranging from (a), full-blown *fiatness* that is exclusively based on convention, to (e), for which we argue that it is actually a *bona fideness*, since it is exclusively based on real properties of a causally dynamic reality. To the latter case belong all those entities that are delimited on grounds of their causal properties and dispositions—functional units, as for instance your right upper arm. Whereas one can argue that the boundaries of such functional units are to some degree fuzzy and indeterminate, this indeterminacy is granularity-dependent like all other *bona fide* boundaries, and one could argue that they can be assigned to conceptual rather than ontological reasons.

A biological object can be looked upon from very different perspectives (e.g. spatio-structural, developmental, physiological, evolutionary), with each perspective putting a different focus on the real properties of the object. As a consequence, when partitioning an object, the resulting partition will differ from perspective to perspective [30]. Whether a given partition will yield *fiat* parts or *bona fide* objects will not only depend on the real properties of the entity and the level of granularity of focus, but also on the perspective applied and thus, to a certain degree, also on the interests of the person conducting the partition.

Unfortunately, many aspects of the ontological theory of *fiatness* have been developed in the context of geographical use cases and applications (e.g. [6]–[10], [17]). Anatomical structures and biological objects in general have been touched upon only briefly and await closer examination in terms of consequences for the criteria of distinguishing *fiat* from *bona fide* boundaries and their status as a categorial ontological distinction (for exceptions see [12], [28], [31]). Since biological entities actively participate in many different types of causal processes as causal agents in a dynamic reality, we will take a closer look at the different types of *dispositions* and *historical relations* that can be used for partitioning and demarcating biological material entities. Biological processes range from evolution to individual development and embryogenesis, from physiological processes within an organism to all kinds of ecological and social interaction. We think that the discussion about what criteria must be used for distinguishing *fiat* from *bona fide* boundaries, and *fiat* from *bona fide* entities, respectively, must not only consider intrinsic spatio-structural properties but also the causal roles and historical relations of material entities and the ontological nature of the respective systems in which they constitute subsystems.

### 2.3 Evidence for the Mind-Independence of some Non-Structural Boundaries: I. Bearers of Function *(Dispositions—Future-Oriented ‘Universal’ Causality)*

In the following we will provide examples of biological entities that, when following BFO's definition, would have to be treated as *fiat* object parts although their boundaries do not rest on any acts of human decision and thus exist independent of any mental or linguistic activities. However, they differ from the usually used examples for *fiat* entities in that the properties used for delineating the entities are not exclusively restricted to intrinsic spatio-structural properties. Instead, they rely on spatio-structurally delimitable *bona fide* landmarks in combination with dispositions (i.e. potential for causal interaction) or historical relations for delineating the corresponding entities. Although the specification of the actual location of the respective boundaries often involves fuzziness, they nevertheless delineate entities that exist independently of all human cognitive acts. In this sense, these entities are therefore truly *bona fide*.

#### 2.3.1 Dispositions Independent of Morphogenesis

Three different basic types of functional units that refer to dispositions that do not involve morphogenesis can be distinguished for biological material entities.

1. **Locomotory dispositions & functional units of locomotion:** Mobile organisms usually possess various parts that are bearers of the disposition to move or to be moved relative to the position of the organism as a whole. Your right upper arm, for instance, is spatio-structurally bounded by a portion of *bona fide* boundary, i.e. the surface of your skin, but also by a portion of *fiat* boundary, i.e. the demarcation from your right forearm and your trunk. According to Smith's [6]–[10] notion of *fiatness*, your right upper arm would therefore be a *fiat* body part. Moreover, following this notion of *fiatness*, its demarcation would exclusively rest on grounds of mental and linguistic activities—your right upper arm would represent an artificially delimited part of your body. The recognition of your right upper arm as a functional unit of locomotion, however, is not exclusively the product of mental and linguistic activities, and its delimitation from the rest of your body is not arbitrary and does not merely rest on acts of human decision. When leaving aside BFO's strictly spatio-structural framework and, instead, employing a framework of functional systems that focuses on the locomotory musculoskeletal system, your upper arm is a *bona fide* entity—a genuine natural (i.e. mind-independent) unit of locomotion that is delimited by its locomotory dispositions. Your right upper arm is a functional unit or element of locomotion that can move or be moved independent from the rest of your body. Granted, purely spatio-structurally, your upper arm is delimited from the rest of your body involving *fiat* boundaries. However, these *fiat* boundaries rest on *bona fide* landmarks, as for instance the proximal and distal limits of your right humerus, which is a *bona fide* object, and are thus also spatio-structurally not completely *fiat* in a strict sense. In fact, they are very close to *bona fideness*. This *bona fideness* is additionally affirmed by the locomotory function of the respective entity.
  In the same way one can argue that your right forearm, your hand and each of your fingers are *bona fide* functional units of locomotion, although they are *fiat* entities from a strictly spatio-structural point of view. The important point here is that their delimitation is not the product of mental or linguistic activities, but reflects reality, only from a locomotory-functional perspective instead of a purely spatio-structural perspective. This can result in spatio-structurally fuzzy delimited *bona fide* entities, as the example of your right upper arm indicates.
2. **Physiological dispositions & functional units of physiology:** Organisms usually possess various parts that are bearers of the disposition to actively participate in physiological processes *within* the organism, which are more or less vital for sustaining the integrity of the organism as a whole, keeping it operating and alive. These parts physiologically *interact* with other parts of the same organism or with ingested biotic and abiotic substances. Spatio-structurally, many of these parts are *fiat* entities, as for instance the human heart, which is delimited from its connecting veins and arteries by *fiat* boundaries. Physiologically, however, due to the functional role the heart has as a pumping organ maintaining the blood flow, it is a *bona fide* functional unit. The active center of an enzyme is another example of a *bona fide* functional unit of physiology that is spatio-structurally a *fiat* entity. Its spatio-structural demarcation involves *fiat* boundaries, which, however, rest upon *bona fide* landmarks (e.g. transmembrane domain, extracellular domain, transition between alpha helix and pleated sheet, coils, folds, indentations, grooves). Functional units of physiology exist independent of human mental or linguistic activities in the same way as functional units of locomotion.
3. **Physiological dispositions & functional units of ecology:** Organisms possess various parts that are bearers of the disposition to actively participate in causal processes involving *parts of other organisms* or material entities from their respective abiotic environment. In other words, every organism possesses structures with which it *interacts* with its biotic and abiotic *environment* and which are vital for sustaining the integrity of the organism as a whole. Because the respective functions of these structures involve a larger surrounding system, including the biotic and abiotic environment, they are ecologically relevant. Spatio-structurally, many of these interacting parts are *fiat* entities, as for instance the eye pits of gastropod species of *Patella*, or the pinhole eye of *Haliotis* species, the abalones, which represent specific concave regions of the epidermis, in which the epithelial cells are differentiated to photoreceptor cells but remain continuously connected to the rest of the epidermis. Although these eyes are spatio-structurally *fiat* entities, due to the functional role they take in for the organism as a whole in interacting with its environment, they are ecologically *bona fide* functional units. Like the other types of functional units discussed above, functional units of ecology exist independent of human mental or linguistic activities.

#### 2.3.2 Dispositions Involving Morphogenesis

Two different basic types of functional units that refer to dispositions that involve morphogenesis can be distinguished for biological material entities.

1. **Morphogenetic dispositions & functional units of development:** Biological objects originate, transform, mutate, merge, and differentiate. They are in a constant flux. The processes of genetically and environmentally induced changes of the spatio-structural composition and qualities of an organism are generally referred to as its development. All parts of an organism bear the disposition to develop. Each part has its own genetically determined developmental sequence of changes, called its morphogenesis. Often, the development of one part is causally dependent on the development of another part or both developments are controlled by the same genetic control mechanism. As a consequence, their development is causally linked and coordinated with one another—they cannot develop independently from each other. In other words, they form a functional unit of development.
  For instance, the distinction of the three germ layers mesoderm, endoderm and ectoderm of undifferentiated tissue in early embryogenesis is the distinction of three functional units of development that can be characterized by the different morphogenetic dispositions they bear. Usually, the three germ layers cannot be differentiated on purely spatio-structural grounds. Despite their spatio-structural *fiatness*, the layers are nevertheless *bona fide* functional units of development, because they exist independent of human mental or linguistic activities. Other examples are the 4 d cell in spiralian metazoans, which gives rise to the entire entomesoderm of these animals, the spermatogonia that gives rise to sperm cells, and the apical meristem in higher plants that gives rise to stem and leaves and their derivates.
2. **Morphogenetic dispositions & functional units of reproduction and propagation:** Organisms have the disposition to reproduce, either sexually or asexually. During the course of evolution, various structures (and behavioral strategies) have evolved that bear the function to facilitate the reproduction of the organism and the propagation of its offspring. These structures form functional units of reproduction and propagation. Typical examples for functional units of reproduction are sexual organs, as for instance flowers, and anatomical structures that attract the attention of potential partners for mating, as for instance the colorful plumage of males in some bird species, which are usually spatio-structurally demarcated from the rest of the organism by *fiat* boundaries. This holds for many functional units of propagation as well, as for instance fruits, the parachutes of *Taraxacum* species, and the hooks of the seed of *Arctium* species. Despite their spatio-structural *fiatness*, these units nevertheless exist independent of human mental or linguistic activities and thus represent *bona fide* functional units of reproduction and propagation.

#### 2.3.3 Structural Anatomy versus Functional Anatomy

The examples above demonstrate that entities can be demarcated not only exclusively on grounds of their intrinsic spatio-structural properties, but also on grounds of a combination of their spatio-structural properties and their dispositions. Whereas the former is important when delineating entities of *structural anatomy*, the latter is important for delineating entities of *functional anatomy* (cf. [31]). The boundaries of entities of structural anatomy are determined on spatio-structural grounds, the boundaries of entities of functional anatomy are determined in such a way that the entity delineated is a function bearer—a unit that, as a whole, bears the disposition to perform a certain function [31]. Entities of functional anatomy, however, often involve spatio-structurally *fiat* boundaries that are only *bona fide* from a functional point of view. Some functional units, as for instance metanephridial systems, even lack physical connectedness and are spatio-structurally discontinuous, because they are composed of a spatially separated group of material entities, i.e. the filtration site formed by podocytes at the blood vessels and the nephridial duct draining the coelomic space that store the filtrate. Instead of physical connectedness, they exhibit functional connectedness.

The identification of entities of functional anatomy often rests on the use of spatio-structural *bona fide* landmarks that are subparts of the entity, and the localization of the entity's boundary usually involves some degree of fuzziness. This fuzziness and indeterminacy, however, cannot be used as a categorial argument against their possible *bona fide* ontological nature, because the respective indeterminacy is in the same way granularity-dependent as the indeterminacy of spatio-structural *bona fide* boundaries at finer levels of granularity (see above).

From the fact that boundaries are not only granularity-dependent but also perspective-dependent, we conclude that for any biological organism one can always distinguish spatio-structural partitions from spatio-functional partitions. Moreover, as the examples from above indicate, several different spatio-functional partitions can be differentiated (e.g. locomotory, physiological, ecological, developmental, reproductive). What is seen as *fiat* and what as *bona fide* depends on the perspective: a given entity can be *fiat* from a spatio-structural point of view, but *bona fide* from a functional point of view. As a consequence, one has to distinguish a taxonomy of structural anatomy from a taxonomy of functional anatomy (see also [31]), or its more specific taxonomies of various types of functional anatomies.

Restricting the criteria for delimiting *fiat* from *bona fide* boundaries to intrinsic spatio-structural properties and ignoring all dispositional properties of an entity reflects the position of the proponents of *form* of the traditional opposition of *form* versus *function* (see the famous controversy between Etienne Geoffroy St. Hilaire and Georges Cuvier [32]–[34]). Hitherto, most ontology authors focused on form rather than function when discussing the ontological nature of boundaries and basic categories of material entity—they propagate, so to speak, Etienne Geoffroy St. Hilaire's position. We want to bring Georges Cuvier's position into the discussion.

### 2.4 Evidence for the Mind-Independence of some Non-Structural Boundaries: II. Bearers of Common Historical Traces *(Historical Relations—Past-Oriented ‘Particular’ Causality)*

#### 2.4.1 Structural Integrity and Stability of an Entity over Time

Three different basic types of historical units can be distinguished that refer to a common causal history that is responsible for maintaining the structural integrity and stability of the respective biological material entities over time. (*Please note that we ignore all questions regarding temporal boundaries of the time of existence of material entities. Their discussion goes beyond the scope of this paper.*)

1. **Developmental relations & historical units of development:** Whereas a functional unit of development is a material entity that is delimited by bearing a specific developmental disposition, i.e. a promise to behave in a certain way in future developmental processes, a historical unit of development, in contrast, is a material entity that is delimited by the fact that all its parts *share the same developmental history*. Thus, the developmental processes have already occurred and certain particular structures shared the same developmental history. A historical unit of development is composed of those particular parts of a particular individual organism that have *proven in the past* that they have developed in concert, as a unit of development, thereby maintaining the spatio-structural integrity of the entity as a whole. In other words, the entity, with all its parts, is delimited on grounds of the fact that it *has acted as a functional unit of development in the past*. Its identity is thus based on a common morphogenetic history and not on morphogenetic dispositions.
  One can, indeed, argue that the defining properties of a historical unit of development, i.e. their shared developmental origin, supervene on the morphogenetic dispositions and thus on the intrinsic functional properties of its corresponding functional unit of development, since the former is an *effect* caused by the latter. Epistemologically, however, when it comes to identifying morphogenetic dispositions and with them functional units of development, one must identify the respective historical units of development that serve as empirical evidence for the existence of morphogenetic dispositions in the first place. Anyhow, just like functional units of development, historical units of development exist independently of human mental or linguistic activities, although in many cases they possess spatio-structurally *fiat* boundaries.
2. **Heredity relations & historical units of heredity:** Whereas historical units of development relate to the integrity of particular parts of a particular organism during its individual development, population biologists also talk about other units that show integrity over time, thereby crossing the spatio-temporal boundaries of individual organisms (e.g. species as individuals, see [35]). Many bio-species or bio-populations, for instance, maintain a high degree of structural integrity or homogeneity of their member organisms over time. And so do genes or morphological traits that exhibit considerable stability within a population over a certain period of time. These structures are historical units of heredity that result from reproductive mechanisms that guarantee a high degree of similarity between the original morphological structure and its copies. And, again, also historical units of heredity exist independently of human mental or linguistic activities, although they in many cases possess spatio-structurally *fiat* boundaries.
3. **Heredity relations & historical units of evolution:** If we take a look at historical units of heredity at a coarser time-resolution, additional mechanisms must be in effect in order to still maintain structural integrity or stability. It requires stabilizing selection pressures for a given inheritable structure to maintain its spatio-structural integrity through an evolutionary period of time. The respective structures form historical units of evolution. For instance the “members” of a phylogenetic character state (i.e. a set of particular inheritable traits that are structurally identical throughout representatives of various species due to homology) are good examples for historical units of evolution (see also *modularity* [36]–[39]). Historical units of evolution also exist independently of human mental or linguistic activities, although they possess in many cases spatio-structural *fiat* boundaries.

#### 2.4.2 Lineages—Constituent Historical Relations of Entities distributed in Time and Space

Whereas the examples discussed above concern material entities that maintain their structural integrity and stability during some period of the lifespan of an organism or even across the spatio-temporal boundaries of single individuals, biological material entities can also historically relate to one another independently of any shared structural stability. When common historical origin is the only defining criterion, the respective sets of structures usually form spatio-temporally scattered groups of material entities whose delineation as a single material entity requires a principle of connectedness (i.e. principle of unity; see also [24]) other than the principles of physical connectedness, like electro-chemical bonds or physical junctions. The example of the functional connectedness of the scattered parts of a metanephridial system mentioned above already demonstrated that it is in principle possible to apply different perspectives for delineating scattered entities, i.e. groups of spatially separated material entities (for *groups* see [25]). Common historical origin may serve as another principle of connectedness that allows the delineation of groups of spatio-temporally scattered material entities. (*Again, please note that we ignore all questions regarding temporal boundaries.*)

1. **Relations of common developmental origin & developmental lineages:** When a cell divides into two daughter cells during a cell fission event, the two daughter cells share the same developmental origin. Although they may migrate to locations separated in space and may proliferate further into separate tissues (e.g. 4 d cell derivates), they nevertheless constitute, together with their parent cell from which they originated and which does not exist anymore, a developmental lineage. Developmental lineages, although spatio-structurally and spatio-temporally delimited by *fiat* boundaries, nevertheless exist independently of human mental or linguistic activities and are in this sense *bona fide* in nature.
2. **Relations of common kinship & genealogical lineages:** Whereas developmental lineages are restricted to the spatio-temporal boundaries of a single organism, the respective structures can be inherited to offspring and thereby constitute genealogical lineages that cross this boundary. In biology we are talking about gene lineages, lineages of morphological traits or even the kinship relations (i.e. parent-child relations) of families of individual organisms. They represent groups of entities that relate to one another across the spatio-temporal boundaries of a particular individual organism, based on common kinship. The phenomenon of infertile castes within some social insects, like for instance the worker caste of ants, demonstrates that these kinship relations can also have a biological impact (i.e. explaining the existence of infertile workers with otherwise zero fitness) and are thus not merely a construct of human mental or linguistic activities. Therefore, also genealogical lineages, although spatio-structurally and spatio-temporally delimited by *fiat* boundaries, nevertheless can be *bona fide* in nature.
3. **Relations of common ancestry/descent & evolutionary lineages:** If we take genealogical lineages to a coarser time scale and allow them to also cross the spatio-temporal boundaries of bio-populations and species, we are looking at evolutionary lineages. When we are talking about homologues, gene families, apomorphic characters and monophyletic groups of species, we are talking about evolutionary lineages. Evolutionary lineages are spatio-structurally and spatio-temporally delimited by *fiat* boundaries, but nevertheless exist independently of human mental or linguistic activities and are in this sense *bona fide* in nature.

#### 2.4.3 Structural Anatomy versus Historical/Evolutionary Anatomy

The examples given above demonstrate that some material entities, be they scattered or not, can be demarcated on grounds of a combination of their historical relations and their intrinsic spatio-structural properties. Their delineation is relevant for *historical/evolutionary anatomy*. The boundaries of historical/evolutionary entities of anatomy are determined in a way that the entity delineated is a whole, either spatio-temporally scattered or connected: every historical/evolutionary entity of anatomy is composed of parts which share the same historical/evolutionary origin, with no entity not belonging to it sharing the same historical origin.

Just like with entities of functional anatomy, historical/evolutionary entities of anatomy often involve spatio-structurally *fiat* boundaries that are only *bona fide* from a historical/evolutionary point of view. Their specification usually involves the use of spatio-structural *bona fide* landmarks of subparts of the entity, and their demarcation often involves some degree of fuzziness as well. While the determination of the boundaries of historical entities is often conducted by observation with the aid of specific instruments (e.g. video tracing, 4D microscopy), the actual determination of the boundary of evolutionary entities is usually the result of an extensive comparison of the spatio-structural properties of various structures and necessarily remains hypothetical.

## Discussion

### 3.1 Boundaries Depend on Granular Perspective

Smith and Varzi [9], [10] claimed that the distinction of *fiat* and *bona fide* is categorial, since it (i) exhaustively covers all possible cases of physical boundaries and (ii) unambiguously draws the line between mind-dependent and mind-independent reality. Moreover, the distinction is ontologically important, because it provides a clear criterion for distinguishing two foundational categories of material entity, i.e. *fiat* and *bona fide* entities, simply on the basis of the type of boundary that the entity possesses. With the examples given above we demonstrate that the distinction is not strictly categorial and that this prevailing view regarding the distinction between *fiat* and *bona fide* boundaries and entities is based on a rather static spatio-structural framework that completely ignores the dynamic nature of reality.

This is insofar unfortunate, as many scientific fields are not interested in purely spatio-structurally defined types of material entities. Instead, they focus on types of entities that *interact with* or *react to* other entities or specific basic conditions, or they are interested in entities that share a specific *history*. These types of entities cannot be characterized solely on spatio-structural grounds. Hence, their boundaries cannot be determined on purely spatio-structural grounds either. As the examples from above also demonstrate, some entities cannot be demarcated within a strictly spatio-structural framework. However, they may nevertheless be demarcated using other frameworks. Many of the entities delineated this way are *not* demarcated merely as a product of mental or linguistic activities, but as entities that reflect a mind-independent reality. Although their boundaries are often spatio-structurally indeterminate and *fiat*, these boundaries nevertheless delineate entities that exist independently of any human cognitive act. Therefore, these entities are in the best sense *bona fide* entities. Their *fiatness* and indeterminacy thereby only concerns the spatio-structural aspects of their reality, but not their defining properties, be they functional or historical.

#### 3.1.1 Granular Partitions and Basic Categories of Boundaries

So far, we have shown that the distinction between *fiat* and *bona fide* boundaries is not as straightforward as it is usually assumed. Instead, we are dealing with a variety of different foundational categories of boundaries that are independent of the distinction between *fiat* and *bona fide* itself: Before one can decide whether a given boundary is *fiat* or *bona fide*, one *first* has to specify from which perspective this distinction is being made. In other words, **the ontological status of a given boundary is perspective-dependent**.

In a series of papers, Smith and coauthors (e.g. [15], [16], [40], [41] have introduced a formal theory of granular partitions and discussed its consequences and implications for various problems of reference and truth, including the abovementioned *Problem of the Many*. Smith and Brogaard argue that whenever we use an expression to refer to some real entity, this brings about *“a partition of reality into two domains: the foreground domain, within which the object of reference is located, and the background domain, which comprehends all entities left in the dark”* [16] (see also [15], [41]). As a consequence, every partition has its granularity built in and is an artifact of perception, judgment or classification [41]. Judgments, at their turn, *“come along with partitions of reality of various sorts, whose type, granularity and scope depend upon the contexts in which our judgments are made”* [16].

Therefore, according to this theory of granular partitions, every partition is judgment-dependent and context-dependent (instead of the term ‘context’ we use the term ‘perspective’, which serves a similar function—we prefer ‘perspective’, because it refers to Keet's formal theory of granularity [42] and we already used it in [26], [30]). A context, at its turn, is of a certain granularity, and it is understood to be *“a portion of reality associated with a given conversation or perceptual report and embracing also the beliefs and interests and background knowledge of the participants, their mental set, patterns of language use, ambient standards of precision, and so forth”* [16]. A context is thus *“a matter of what is paid attention to by participant speakers and hearers on given occasions”* [16]. Since granular partitions are context-dependent, a granular partition is *“a device for focusing upon what is salient and also for masking what is not salient”* [16]. As a consequence, a granular partition that puts a particular entity into its foreground will not necessarily recognize all the entity's parts and subparts: when a math teacher counts the number of students in her class, she will use a partition that does not recognize the students on the level of their cellular and subcellular composition. Accordingly, when she has to grade the students' performance in her class, she will use a partition that only judges their performance in math and not in English.

The theory of granular partitions can also manage vague boundaries: *“vagueness de dicto is captured at the partition level via multiple ways of projecting crisply”* [15] (‘projection’ here refers to the relation from judgment to reality, i.e. the relation from a partition cell to its corresponding portion of reality [40]). A granular partition is considered to be *crisp* if it projects onto reality in a single and unique manner, whereas it is considered to be *vague* if it involves a multitude of projections onto reality, thereby interpreting vagueness as a semantic property of names and predicates [15]. Thus, the theory of granular partitions introduced by Smith and coauthors provides an adequate formal framework for dealing with the problem of vague and indeterminate boundaries.

Moreover, in combination with Keet's formal theory of granularity [42], the theory of granular partitions also provides the formal framework for dealing with problems of the granularity-dependence and perspective-dependence of boundaries. Thereby one should note that every partition by itself is entirely fiat in nature [16]. However, as Smith and Brogaard argue, some partitions *“are coordinated with bona fide demarcations on the side of objects in reality and some of them merely with fiat demarcations which we ourselves have introduced into reality in our various dealings with nature”* [16]. Smith and Brogaard thus distinguish partitions that track *bona fide* boundaries in reality from partitions that track boundaries that only exist as a result of acts of human fiat. Unfortunately, Smith and coauthors do not provide any criteria for distinguishing *fiat* and *bona fide* boundaries—they merely discuss the possibilities of *fiat* and *bona fide* partitions.

The hitherto prevailing notion of boundaries restricted itself to the spatio-structural perspective that does not account for the role of time and thus processes in reality and how they affect the ontology of boundaries and the ontology of basic types of material entities. In other words, **the strictly spatio-structural perspective is ignorant of causality**. Above we have shown that there are other types of entities besides purely spatio-structurally defined ones, all of which are epistemologically relevant and empirically accessible. The main difference between them and spatio-structurally defined entities is that only spatio-structural entities are exclusively demarcated on grounds of their intrinsic structural properties, their qualities. In contrast, the other entities are defined either (i) in terms of dispositions and therefore involve types of processes that are *repeatable* in principle (i.e. based on a notion of *universal causality*), or (ii) they are defined in terms of historical relations and therefore involve a particular sequence of processes that *has taken place in the past* and the effects this sequence had on a collection of particular spatio-structural entities (i.e. based on a notion of *particular causality*). The resulting general distinction of foundational types of boundaries and of material entities thereby reflects the very basic distinction of scientific disciplines or aspects of empirical sciences, which can be classified into the following three basic categorial perspectives (i.e. context categories):

1. ***Spatio-structural perspective***: the view on what *is given* now, at a particular point in time, i.e. what is *intrinsically inherent* in material entities; *descriptive* and inventory-oriented; restricted to passive observation; represents reality in a way that is analog to the painting of a still life (cf. Etienne Geoffroy St. Hilaire's structuralism position of priority of *form*).
2. ***Predictive perspective***: the view on what *can happen* in the future, dealing with dispositional/functional aspects of reality and thus with *potentiality*; *predictive* and systems-oriented; involves experimentation and the active interference and manipulation of a human-independent reality by an investigator; represents reality as a dynamic system and describes material entities with a focus on their potential future interactions, i.e. models an entity's causal space of possible (inter-)action (cf. Georges Cuvier's functionalism position of priority of *function*).
3. ***Retrodictive (diachronic) perspective***: the view on what *has happened* in the past, *retrodictive* and history-oriented; involves the observation and description of particular processes or the reconstruction of past processes by comparing present distribution patterns of spatio-structural properties and their bearers; represents reality as a dynamic system and describes material entities with a focus on their historical interactions and common origins (Karl Ernst von Baer's position of *embryology*; Charles Darwin's position of *evolution*).

Above, we have argued that one can meaningfully distinguish *fiat* from *bona fide* boundaries and *fiat* from *bona fide* entities within all three perspectives. In all three perspectives *fiatness* implies mind-dependent (i.e. purely epistemological/conceptual) delimitation, whereas *bona fideness* implies mind-independent (i.e. natural, ontological) delimitation. As a consequence, some entities are spatio-structurally demarcated by *fiat*, although functionally they are *bona fide*. One can argue that these spatio-structurally *fiat* boundaries are nevertheless mind-independent and, as a consequence, *bona fide*, but only from a non-spatio-structural point of view. Therefore, one must distinguish between mind-dependent and mind-independent spatio-structurally *fiat* boundaries.

Our analysis implies that if one does not restrict the distinction of *fiat* and *bona fide* boundaries to a purely spatio-structural framework, but, instead, focuses on the less restrictive and thus more general framework of mind-dependence and mind-independence, several different categories of *fiat* and *bona fide* boundaries must be distinguished. Our examples demonstrate that we should not talk about *fiat* and *bona fide* boundaries without specifying the perspective we use, because many biological objects can be partitioned into *bona fide* components in various different ways. Consequently, the distinction of *fiat* and *bona fide* material entities necessarily depends on the perspective as well (for a discussion of *granularity perspectives* cf. [42], [30]).

Any perspective that itself is *mind-independent* provides a *principle of identity* (cf. [24]) that depends on the specific aspects of reality that are taken into account (i.e. intrinsic spatio-structural properties versus functional dispositions versus historical integrity or historically caused distribution patterns) and has its own two categories of boundaries, *fiat* and *bona fide* respectively. Therefore, one has to distinguish spatio-structural from functional and historical boundaries, and thus also spatio-structural from functional and historical types of material entities. This results in a taxonomy of spatio-structural entities alongside a taxonomy of functional entities and a taxonomy of historical entities. Moreover, in case one further differentiates these foundational perspectives into several more specific perspectives that are still ontologically independent from each other (i.e. perspectives must not supervene on each other), it results in even more taxonomies. This is the case for the different types of functional entities, i.e. locomotory, physiological, ecological, developmental, and reproductive entities, with each corresponding perspective resulting in its own specific taxonomy of material entities.

It should be noted that distinguishing types of boundaries in terms of *fiatness* versus *bona fideness* and at the same time in terms of different mind-independent perspectives results in the recognition of distinctions that are ontologically independent from each other and thus truly categorial. Muscles of multicellular animals, for instance, usually can be clearly delineated and distinguished from each other on grounds of their spatio-structurally *bona fide* boundaries. This is not necessarily the case when employing a locomotory perspective, since sometimes several muscles are innervated by the same nerve and thus cannot be moved independently from each other. As a consequence, at least from a locomotory perspective, not every individual muscle does constitute a *bona fide* functional unit of locomotion. Instead, individual muscles exist that are functionally *fiat* entities of locomotion. Thus, some spatio-functionally *fiat* entities are spatio-structurally *bona fide* objects, and vice versa, which demonstrates the ontological independence of the respective categories and their underlying perspectives.

### 3.2 Consequences for Ontology Design

We have shown that the distinction of *fiat* and *bona fide* boundaries and *fiat* and *bona fide* material entities is not only granularity-dependent, but also perspective-dependent. It is important to realize that the perspective-dependence does not imply a representational arbitrariness. The perspective-dependence of the distinction of *fiat* and *bona fide* boundaries and *fiat* and *bona fide* material entities does not merely result from human cognitive acts, but also depends on the entities themselves. As Smith and Brogaard point out: *“while partitions, and the cells by which they are constituted, are artefacts of our cognition, when once a given partition exists, it is, for each cell in the partition and for each object in reality, an objective matter whether or not that object is located in that cell”* [16]. There are *bona fide* partitions that correlate with reality. This is the case, because the foundation for the distinction of *fiat* and *bona fide* is the distinction of mind-dependent and mind-independent delimitations, independent of the perspective. Therefore, by differentiating several types of *fiat* and *bona fide* boundaries and allowing a material entity to be at the same time *fiat* and *bona fide*, we do not relativize the categorial distinction of *fiat* and *bona fide* in the sense that it would not represent a distinction that has a real correlate in nature. Instead, we rather point to the fact that the distinction of foundational types of boundaries has to be further differentiated, in order to accommodate additional perspectives into the ontological consideration besides the spatio-structural one.

What are the consequences for ontology design? Unfortunately, an already well known problem of ontology design becomes even worse: reality cannot be modeled within a single, universal and ontologically consistent taxonomy of top-level categories of material entity that can be easily organized within a universal single-inheritance tree (see also [26]). Considering ciliated and rhabdomeric light sensory cells as an example: should the category ‘ciliated light sensory cell’ be organized as a subcategory of ‘ciliated cell’ or as a subcategory of ‘light sensory cell’? We are dealing with the lineup of intrinsic spatio-structural qualities on the one hand and functional dispositions on the other hand. Which one should we give preference in a universal single-inheritance taxonomy? Another example would be ‘mesodermal muscle cell’, which could be organized either as a subcategory of ‘muscle cell’ or as a subcategory of ‘mesodermal cell’. Here, a structural-functional property (i.e. muscle) is lined up against a developmental property.

The situation for ontology design is somehow comparable to the problem of how to best organize and represent the system of chemical elements. With the periodic table, Dmitri Mendeleev came up with a good solution for the latter, in which he combined two very simple and ontologically independent but each other overlapping taxonomies within a single table. If we would only have to deal with ‘ciliated light sensory cell’, we could use the same approach in biology and combine a spatio-structural with a functional taxonomy, resulting in a hierarchical table of biological entities. However, as the second example, ‘mesodermal muscle cell’, and the other examples given in this paper indicate, in biology we are dealing with more than two categorial perspectives and thus more than two foundational taxonomies of different categories of material entity and, therefore, a tabular organization like the periodic table is not applicable.

The necessity to distinguish different taxonomies of material entity results from the perspective-dependence of boundaries and the existence of a multitude of ontologically independent perspectives. Which perspective is considered to be relevant thereby usually depends on the specific interests of the researchers and thus on a specific discipline or domain. Formal top-level ontologies must be compatible for all kinds of relevant scientific interests and thus must accommodate various perspectives. Therefore, and in order to guarantee the comparability and compatibility of different application and domain reference ontologies, formal top-level ontologies must provide a consistent and uniform template of how these different taxonomies and their respective top-level categories must be organized.

The Basic Formal Ontology (BFO; http://www.ifomis.org/bfo; [11]) is such a scientific formal top-level ontology. In BFO, each category has exactly one single asserted parent class (except for the root category). This is the result of BFO having been developed according to the *single inheritance policy*, which requires all defined categories to be disjoint and exhaustive, i.e. categories must be mutually exclusive relative to a given level of granularity [43]. An important question that results from our findings is how to organize multiple, ontologically independent taxonomies within a single universal single-inheritance taxonomy?

Because every unit, independent of whether it is a developmental, ecological, evolutionary or physiological unit, necessarily possesses spatio-structural properties, the spatio-structural taxonomy can be used as backbone taxonomy: it is possible to classify the leaf categories of every non-spatio-structural taxonomy within the spatio-structural taxonomy according to their spatio-structural properties. As a consequence, leaf categories must be defined in reference to both spatio-structural properties *and* their specific defining dispositions or historical relations. Following this approach, the category ‘ciliated light sensory cell’ would be defined as:

*A ciliated light sensory cell is a ciliated cell that is light-sensitive*.

As such, ‘ciliated light sensory cell’ is a direct subcategory of the spatio-structural category ‘ciliated cell’. However, it is at the same time also a direct subcategory of the physiological category ‘light sensory cell’. Two questions immediately emerge: (i) where to place the category ‘light sensory cell’ within the spatio-structural taxonomy and (ii) how to specify the parent-child relation between ‘light sensory cell’ and ‘ciliated light sensory cell’ without violating the single inheritance principle?

The first question translates into the question of what spatio-structural properties do all instances of ‘light sensory cell’ have in common, which will specify the location of ‘light sensory cell’ within the spatio-structural taxonomy? This is not trivial, since new types of light sensory cells might be discovered in future, which would change the position of ‘light sensory cell’ within the spatio-structural taxonomy. We only know for certain that every light sensory cell necessarily is a cell and that it is necessarily light-sensitive. Thus, we could define ‘light sensory cell’ as:

*A light sensory cell is a cell that is light-sensitive*.

However, when comparing the direct functional child categories of ‘light sensory cell’ we might discover that we can provide a more specific spatio-structural definition. After all, light sensory cells must possess some light-sensitive proteins (i.e. opsin; e.g. [44]), which can help to identify a putative light sensory cell on purely spatio-structural grounds (e.g. by *in situ* hybridization of expressed genes; [45]). One could thus distinguish between a conservative part of the definition of ‘light sensory cell’ that is independent of its current composition of child categories (i.e. *a light sensory cell is a cell that is light-sensitive*) and a dynamic part that directly results from and thus depends on the comparison of spatio-structural features of all its child categories (i.e. *a light sensory cell is a cell that possesses some light-sensitive protein and that is light-sensitive*). Whenever a new child category is added, the dynamic part may change accordingly.

Regarding the second question of how to specify the parent-child relation between ‘light sensory cell’ and ‘ciliated light sensory cell’, we must introduce additional ontology relations for consistently organizing the various taxonomies and their interrelationships within a single universal single-inheritance taxonomy. For instance the transitive (i.e., for all *A_i_* holds, if *A_1_* is-a *A_2_* and *A_2_* is-a *A_3_*, then *A_1_* is-a *A_3_*) ‘is-a’ relation for class-subclass relationships should be differentiated into for instance a ‘is-structurally-a’, ‘is-physiologically-a’, ‘is-locomotory-a’ and ‘is-ecologically-a’ relation, in order to differentiate the class-subclass relations of the different taxonomies and to allow to still organize them within a single-inheritance tree. In the same way one should distinguish different types of parthood relations as well (e.g. ‘ecologically-fiat-part-of’).

If we want to identify functional and historical *bona fide* entities that are spatio-structurally *fiat*, we also require properties such as ‘ecological-unit’ or ‘developmental-unit’ with a Boolean value space (i.e. yes/no). In case we want to distinguish spatio-structurally scattered units from units that are spatio-structurally connected (i.e. no part of the unit is spatially separated from the rest of the unit by a gap), a property such as ‘ecological-unit’ must be differentiated into the properties ‘ecological-object-unit’ for spatially connected units and ‘ecological-group-unit’ for spatially scattered units (for *groups* see [25]). Moreover, if we want to indicate that a spatio-structurally *bona fide* entity is a *fiat* unit, as it is for instance the case with some muscles that are not innovated individually but only as a muscle bundle, a property as for instance ‘locomotory-fiat-unit’ is required.

Except for lineages, all the different taxonomies discussed above could be organized within a single universal single-inheritance taxonomy for a scientific formal top-level ontology such as BFO. In case of lineages, however, we require ontology relations that indicate the respective historical relation between instances and spatio-structural categories of material entity, as for instance a symmetric (i.e., for all *A_i_* holds, if *A_1_* is-homologues-with *A_2_*, then *A_2_* is-homologues-with *A_1_*) relation ‘is-homologues-with’.

### 3.3 Conclusions

With various examples from biology we have demonstrated that the hitherto prevailingly used criteria for distinguishing *bona fide* from *fiat* physical boundaries are not unambiguously applicable. The ambiguity results from the combined use of two ontologically independent types of demarcation criteria: (i) intrinsic qualitative criteria (i.e. spatial/physical discontinuity, qualitative heterogeneity) and (ii) the criterion of mind-independence. Our examples demonstrate that in many cases physical boundaries are *bona fide* with respect to one type of criterion while *fiat* with respect to the other. Moreover, they demonstrate that the distinction of *fiat* and *bona fide* material entities is perspective-dependent. As a consequence, the distinction of *bona fide* and *fiat* boundaries itself is also perspective-dependent and thus cannot be categorial and absolute. Our examples also demonstrate that if the two types of demarcation criteria for boundaries both must be met, the distinction of *bona fide* and *fiat* boundaries is not exhaustive, because boundaries exist that only meet one type of criterion.

One possible solution would be dropping the criterion of mind-independence and confining the distinction of *bona fide* and *fiat* boundaries merely to spatio-structural criteria. This, however, would do no justice to the existence of the multitude of ontologically independent perspectives and the perspective-dependence of the distinction of *fiat* and *bona fide* material entities.

We therefore concluded that *fiat* and *bona fide* boundaries must be distinguished based on the criterion of mind-independence. The criterion of mind-independence, at its turn, seems to depend on the question whether the entity delimited by the boundary in question is a *bona fide* material entity, i.e. a spatio-structurally bound object or a functionally or historically bound causal unit. In case it is, the respective boundary exists independent of any human cognitive acts and is *bona fide* in this sense. In other words, the ontological status of a boundary depends on the type of material entity it bounds and not vice versa. As a consequence, the distinction of *fiat* and *bona fide* material entities cannot rest on the type of boundary they possess, but must depend on criteria of causal unity—either spatio-structural or predictive or retrodictive (see the main perspectives discussed above).

If the defining criteria for distinguishing *bona fide* and *fiat* entities do not refer to boundary types, one can simplify the distinction of boundaries into those that bound *fiat* entities and those that bound *bona fide* entities, be they spatio-structural objects or functional or historical units. It seems as if the development of BFO 2.0 is currently taking a step towards this direction. In a draft that is currently (Aug. 2012) available from the BFO website (*Basic Formal Ontology 2.0 - Draft Specification and User's Guide*; http://ncorwiki.buffalo.edu/index.php/Basic_Formal_Ontology_2.0), objects are characterized as *natural units* that are *causally relatively isolated* entities, which means that they are structured through some *causal unity*, to which they are maximal (i.e. a maximal causally unified material entity). According to this draft, causal unity thereby includes unity through physical covering, internal physical forces or engineered assembly of components. According to the BFO 2.0 draft, however, this notion of objects still awaits a formal theory. It remains to be seen where the development of BFO 2.0 will finally lead to.

Regarding basic types of boundaries, the draft does not distinguish *bona fide* and *fiat* boundaries anymore, but treats all boundaries of material entities as *continuant fiat boundaries*. Although this notion follows, at least from an epistemic point of view, directly from the theory of granular partitions, which claims that every partition is *fiat* and thus every boundary is *fiat* too, it does not do justice to the underlying ontological nature of the entities bound: we can still distinguish between boundaries which bound entities that correlate with natural units and those which bound *fiat* entities—after all, the BFO 2.0 draft itself still distinguishes between object and *fiat* object part, and if objects are natural units than their boundaries must be natural as well. Therefore, if one wants to distinguish boundaries of different categories of material entities, one could distinguish for instance continuant *fiat* boundary*_Object_* from continuant *fiat* boundary*_fiatObjectPart_* and continuant *fiat* boundary*_locomotoryUnit_* or continuant *fiat* boundary*_ecologicalObjectUnit_*.

We have shown that the problems resulting from the perspective-dependence of boundaries do not only affect very special and highly differentiated categories of material entity, which may be restricted to leaf classes of domain reference ontologies and thus to aspects of reality that are not relevant to formal top-level ontologies. Quite the contrary, they do apply already at the root of the top-level categories of ‘material entity’ and thus must be considered by any formal top-level ontology for the scientific domain, as for instance the BFO.

Unfortunately, the consequences make ontology design more complicated. However, they result from an attempt to meet the requirements of the perspectivalist position that a plurality of alternative perspectives on reality do exist that are ontologically equally legitimate. Apparently, reality is very complex and the problems that we face when organizing and categorizing reality seem to reflect the epistemic constraints and limitations that are inherent to our human cognitive devices when attempting to represent this reality within a consistent mental model. Obviously, we have fundamental problems to comprehend reality within a single universal perspective and therefore have to resort to the epistemic means of multiple perspectives.
